# Exploits in Medicine

**Published:** 1891-10

**Authors:** 


### EDITORIAL DEPARTMENT.

F. E. DANIEL, M. D., EDITOR AND PUBLISHER.

THIS JOURNAL, by unanimous vote of the members, was made the Official Organ of the Austin District Medical Society.

COLLABORATORS:

*Dr. R. M. Swearingen, Austin.*
*Prof. B. E. Hadra, M. D., Galveston.*
*Prof. Geo. Cupples, M. D., San Antonio.*
*Dr. T. C. Osborn, Cleburne, Texas.*
*Dr E. J. Doering, Chicago.*
*Dr. E. J. Beall, Fort Worth.*
*Dr Odo Betz, Germany.*
*Prof. W. B. Rogers, M. D., Memphis.*
*Dr. J. W. McLaughlin, Austin.*
*Dr. T. J. Tyner, Austin.*
*Prof. J. F. Y. Paine, M. D., Galveston.*
*Dr. R. H. L. Bibb, Mexico.*
*Dr. T. J. Bennett. Austin.*
*Dr. Bat Smith, Wharton.*
*Dr. E. Meierhof, New York.*
*L. H. Luce, M. D., West Tisbury, Mass.*

### EXPLOITS IN MEDICINE.

Deeds of daring and chivalry are commemorated in song, and illumine the pages of history: heroes of great battles,—those who "seek the bubble, reputation, at the cannon's mouth," or perish by flood and field, live in the memory of man. Poets, sculptors and orators have their praises sung, and grow immortal.

But who shall chronicle the exploits of the daring doctor? Who sing his praises?

There be those who, jealous, lest posterity should be cheated, or that contemporaneous historians may not do them justice, essay to do, and do their own chronicling; sing their own praises; and, alas, too frequently, do it in very bad English. The average medical journal of the day abounds in narratives; accounts of marvelous cures, wherein the narrator figures in capital letters, so to speak, and the reader loses sight of the details, in admiration of that omnipresent personal pronoun first, singular, the capital "I."

These narratives be, some, amusing, some astonishing, and a few, disgusting. There be those who, in thus playing historian to themselves, become so enthused, we will say, that, like the poet, they take license; they sacrifice veracity, we much fear, to vanity. They think it is impossible to be impressive without exaggeration, and deal exclusively in superlatives. Some are so absorbed in the scientific (?) aspect of their reports that they ignore the ordinary rules of rhetoric, disregard orthography, and flout punctuation, utterly. The meed of praise so richly deserved (in their own minds) for such and such deeds of medical or surgical *prowess*, would not be bestowed by a grudging profession, they argue—did it not exceed all other deeds,—break the record, so to speak; hence, some journal articles we read might well be called "medical Munschausenry." There is about them, something of *bombast* which makes the reader forget the victim—patient, we should say, and think only of how great a doctor that doctor is, *anyhow!*

We read in the *Western Medical Reporter* of July, page 160, and copy verbatim a portion of the chronicle of a mighty exploit in medicine, or rather two chronicles of two mighty exploits by one and the same man. One of these reports is the case of a young woman who was burned "while making apple butter," a fact which the author duly chronicles, but which is not shown to bear any relation to the extent of the burn, or to the cure, the credit of which latter seems to be divided between goose-grease, oiled paper and the doctor; a remarkable case. A burn of half the surface of the body, denuding the spinous processes, (?) destroying the finger nails, etc., and yet after the first few moments, is not attended with shock; nor with nervous prostration, nor delirium, nor fever, nor septicæmia, nor, barring temporary loss of vision, with any constitutional disturbance whatever, and cured by local measures alone.

The other exploit was the report of a case of typhlitis, apparently, from the description given, in a boy of 14. According to the chronicler the boy had "been eating heavy" [sic] and was attacked with vomiting and purging, accompanied, of course, by "crampy pains" and followed by tenesmus. This occurred on Tuesday midnight, 22nd July. This doctor saw him Friday morning and found him with a "pinched, anxious expression—pulse 115, temperature 103, skin hot and dry, respiration quick, tenderness over ileo-cæcal region;" found what he "took to be a tumor—rather indistinct but pressure on it elicited great pain; tympanitis slight."

Failing to "move the bowels by glycerine injections high up, and castor oil"—the Doctor says:

"I put him on morph. gr. ½ and atropine $\frac{1}{75}$ gr. hyperdermically" [sic] "and *held him there* by repeating this as necessary." * * * * ! ! [a 14 year old boy.]

The Doctor proceeds:

"Sunday a. m. saw he was passing into a collapsed condition, pulse nearly gone, skin cold, stupor deepening. Sunday p. m. took a brother practitioner down who agreed with me in diagnosis and gave the boy 48 hours to live. I thought myself the time was plenty long. Tympanites by Sunday was quite pronounced. Monday morning found him as near dead as a person could be and be alive; abdomen so distended he could not breathe except when propped up with pillows, skin perfectly cold. I had asked to perform laparotomy, parents declined, so I decided to see what I could do with a stomach pump inverted; this was spoken of the afternoon before. The only tube I could get was a fountain syringe tube and I lubricated this with goose grease and passed it in about 2½ feet, could get it no farther. Then I used this instrument as follows. [Description omitted.—Ed.] No result at 4 p. m. that day, Monday, five days after first pain, I again passed the tube and I found I could pass it little over 4 feet. There seemed to be less resistance to passing of tube, probably because patient was more moribund. I felt an obstruction to its further progress. Patient told me he could feel end of tube on right side lower down, so I concluded I was somewhere near the seat of trouble. I put on firm pressure, but could not throw in much fluid; while I was thus forcing water in, a distinct report was heard all over the room and persons in the next room heard it. I thought at once that I had bursted [sic] his bowel by over-distention. I dropped the syringe and went to his pulse to watch for total collapse, but it did not come. After waiting five minutes I went again to the syringe and imagine my surprise to find the resistance to the forcing of water had disappeared. [*Now*, we suppose he passed the tube clear through him, punched him out.] I immediately reversed the engine and to hear the air come out was indeed music to my soul. Relief came at once. I threw in glycerine and water and had a movement of bowels soon. The recovery was slow; food liquid, and after bowels had moved gave enemata of water which seemed to draw wind thoroughly." [*Verbatim, literatim et punctuatim.*—Ed.]

Thus is chronicled an exploit of no mean proportions; thus goes on record for posterity, a tale of a daring doctor and an impervious boy,—and, we might say, an indestructible boy. The insurance men might safely accept him as a risk; lightning wouldn't kill him. While the doctor, to gratify an admiring and expectant public, should take the lecture field, or go in a dime-museum. For, surely, a man who can pass a rubber syringe-tube four feet through a winding intestinal tube some twenty odd feet in length, and who can make water "draw wind," would, himself, "draw," anywhere.

It were supererogation to point out the beauties of the above composition; but we *must* say—"more moribund" *is* good; it is as if to say—"more dying,"—"deader." And to raise that boy —after transfixing him,—after he had "bursted," him, and after

"½ gr. morphia and $\frac{1}{75}$ gr. atropine hy*per*dermically" had been repeated, is, indeed an exploit.

In the fullness of our admiration—as Miriam of gladness—we are tempted to burst forth into song; or, like Silas Wegg—the immortal,—drop into poetry:—How's this—

A MODERN WATER-LOO.

There was a sound of vomiting by night;
An Illinois chap was 'rastling then
With a case of cholera morbus. Bright
Was the Doctor, and equal to the emergency;
The boy "had been eating heavily."—When
Th' abdomen rose with its tympanitic swell
A soft rubber tube with goose grease oiled well
Was sent down to investigate; with cunning skill
And a reversible stomach pump the Doctor filled
That boy with aqua pura; and still
All went as merry as a marriage bell.
But hush, hark, a deep sound strikes like something had "bursted"!
Did ye not hear it? No, 'twas but his wind,—
Or the stomach pump rattling o'er the umbilicus;—
On with the picnic,—let th' flatus be unconfined,
Nor sleep till morn; when a case like this I meet,
I hasten to the nearest journal with my (f)lying feat. (B)IRONIC.

Seriously: there is too much of this sort of so called "reports of cases"; and we ask,—what good can come from the publication of such exploits? Such as the cases cited, are calculated to bring reproach upon the profession, and medicine into disrepute. Bad enough, they would be, even if correctly written; but the composition shows as great a lack of knowledge of the English language as of modesty and regard for veracity.

